# Effects of Oxygen Modification on the Structural and Magnetic Properties of Highly Epitaxial La_0.7_Sr_0.3_MnO_3_ (LSMO) thin films

**DOI:** 10.1038/s41598-020-60343-5

**Published:** 2020-02-27

**Authors:** Shalini Kumari, Navid Mottaghi, Chih-Yeh Huang, Robbyn Trappen, Ghadendra Bhandari, Saeed Yousefi, Guerau Cabrera, Mohindar S. Seehra, Mikel B. Holcomb

**Affiliations:** 1 Department of Physics & Astronomy, West Virginia University, Morgantown, WV 26506 USA; 2 Mechanical and Aerospace Engineering, West Virginia University, Morgantown, WV 26506 USA

**Keywords:** Physics, Materials science

## Abstract

La_0.7_Sr_0.3_MnO_3_, a strong semi-metallic ferromagnet having robust spin polarization and magnetic transition temperature (*T*_C_) well above 300 K, has attracted significant attention as a possible candidate for a wide range of memory, spintronic, and multifunctional devices. Since varying the oxygen partial pressure during growth is likely to change the structural and other physical functionalities of La_0.7_Sr_0.3_MnO_3_ (LSMO) films, here we report detailed investigations on structure, along with magnetic behavior of LSMO films with same thickness (~30 nm) but synthesized at various oxygen partial pressures: 10, 30, 50, 100, 150, 200 and 250 mTorr. The observation of only (*00* *l*) reflections without any secondary peaks in the XRD patterns confirms the high-quality synthesis of the above-mentioned films. Surface morphology of the films reveals that these films are very smooth with low roughness, the thin films synthesized at 150 mTorr having the lowest average roughness. The increasing of magnetic *T*_C_ and sharpness of the magnetic phase transitions with increasing oxygen growth pressure suggests that by decreasing the oxygen growth pressure leads to oxygen deficiencies in grown films which induce oxygen inhomogeneity. Thin films grown at 150 mTorr exhibits the highest magnetization with *T*_*C*_ = 340 K as these thin films possess the lowest roughness and might exhibit lowest oxygen vacancies and defects. Interpretation and significance of these results in the 30 nm LSMO thin films prepared at different oxygen growth pressures are also presented, along with the existence and growth pressure dependence of negative remanent magnetization (NRM) of the above-mentioned thin films.

## Introduction

The miniaturization of devices to increase speed and reduce the cost of materials is an ongoing need for many current and potential magnetic technologies such as spintronics, highly dense non-volatile memory, spin-caloritronics, and different multifunctional micro and nanoscale devices^1–5^. Among important materials for device applications are room temperature ferromagnetic oxides such as the manganites, La_1−x_Sr_x_MnO_3_, since their properties can be tuned using a number of degrees of freedom including charge, spin, orbital, and magnetic ordering phenomena^6,7^. They are also excellent candidates for novel engineered nanostructures as they exhibit colossal magnetoresistance (CMR) effect which has been used for different multifunctional applications^8–10^. La_1−x_Sr_x_MnO_3_ also shows CMR phenomena under smaller external magnetic fields compared to the other manganites and strongly correlated materials^11,12^.

Among the La_1−x_Sr_x_MnO_3_ manganites, the optimized stoichiometry La_0.7_Sr_0.3_MnO_3_ (LSMO) is one of the most prominent members with outstanding magnetic and magneto-transport properties. Compared to other typical magnetic oxides, LSMO exhibits semi-metallic behavior, outstanding ferromagnetism, high Fermi-level spin polarization, with a bulk magnetic ordering temperature, *T*_C_ (= 370 K) well above room temperature^13,14^. Also, the spin polarization of LSMO can be enhanced up to ~95 %, with a magnetoresistance (MR) proportion of greater than ~1800 % has been achieved ~4 K^15^. In addition, LSMO films have been widely utilized in giant tunneling MR in magnetic oxide tunnel junction-based devices with effective spin injection behavior in spin valve device structures. In LSMO, strong coupling between lattice-charge-spin provides wide range control of electronic and magnetic transport properties by optimized growth and external actuation.

For device applications, thin film growth of LSMO by molecular beam epitaxy (MBE) or pulsed laser deposition (PLD) is often required. However, highly epitaxial synthesis of lowest roughness single crystal LSMO films having superior physical functionalities has been quite challenging^16^. In the past work, many different approaches have been applied to test the effect of primarily one parameter (i.e. strain, symmetry, oxygen vacancies, La/Sr ratio, etc.) on the measured properties. For example, to test the effect of oxygen vacancies, La_0.8_Sr_0.2_MnO_3_ was grown in a variety of oxygen pressures and different post annealing conditions^17^. It was found that low oxygen pressure during growth degrades magnetic properties, while post-annealing in high oxygen pressure does not recover the magnetism showing that the effect of oxygen vacancies in this strongly correlated system is not trivial^18,19^.

The transport and magnetic behavior of LSMO are very much dependent on the lattice- charge- spin couplings, which can be significantly affected by the growth parameters such as temperature of the substrate during deposition, oxygen growth pressure, substrate to target distance, laser fluence, etc^19–21^. In transition metal oxides, oxygen coordination surroundings of transition metals usually underpin a wide range of functional properties via magnetic interactions and crystal field splitting. Manipulating the oxygen coordination environment mainly in oxide materials is rather difficult as it needs the modification of oxygen-cation bond lengths and angles that can be manipulated only by changing the temperature and applying physical or chemical pressure. La_1−x_Sr_x_MnO_3_ can be represented using Kroger-Vink notation^22^ given in Eq. (1):

1 $$SrO+\frac{1}{4}{O}_{2}+{(L{a}_{L{a}^{3+}}^{3+})}^{x}+{(M{n}_{M{n}^{3+}}^{3+})}^{x}=\frac{1}{2}L{a}_{2}{O}_{3}+(S{r}_{L{a}^{3+}}^{2+})\,{\rm{{\prime} }}+{(M{n}_{M{n}^{3+}}^{4+})}^{\cdot }$$

Here ${(M{n}_{M{n}^{3+}}^{4+})}^{\cdot }$ is represented as a hole. While doping concentration (Sr) is constant, ${(M{n}_{M{n}^{3+}}^{4+})}^{\cdot }$ number also, depending on the concentration of oxygen of thin films. LSMO thin films grown under stoichiometric oxygen pressure loses oxygen according to Eq. (2):

2 $$2{(M{n}_{M{n}^{3+}}^{4+})}^{\cdot }+{({O}_{{O}^{2-}}^{2-})}^{x}=\frac{1}{2}{O}_{2}+2{(M{n}_{M{n}^{3+}}^{3+})}^{x}+{({V}_{{O}^{2-}})}^{\cdot \cdot }$$

The above equation indicates that formation of single oxygen vacancy (OV) occur with the expense of two holes ${(M{n}_{M{n}^{3+}}^{4+})}^{\cdot }$. Hence, the OVs ${({V}_{{O}^{2-}})}^{\cdot \cdot }$are the important defects in LSMO thin films grown at lower oxygen partial pressure^23^. Perovskite materials such as LSMO treated under reduced conditions possess oxygen vacancies which are expected to lower the Mn^4+^ concentration and significantly affect the crystal lattice and physical functionalities.

Considering the importance of the LSMO thin films for both technology and fundamental science, it is necessary to know precisely the magnetic and electronic behavior at different growth conditions for achieving enhanced efficiencies. Since studies on the oxygen growth pressure dependence of structural, magnetic and other physical properties of epitaxial LSMO films synthesized utilizing PLD are quite limited^20^, a detailed investigation on the effect of optimized oxygen deficiency on structural, morphological, and magnetic properties is necessary for the optimization of oxygen vacancies to get best physical properties. In the research reported here, we bring together high-quality film growth and detailed studies on the effects of optimized oxygen growth pressure on the structure and magnetic behaviors of LSMO thin films synthesized by optimized PLD. Results are presented on the detailed investigations on structure, along with magnetic behavior of LSMO films with same thickness (~30 nm) but synthesized at various oxygen partial pressures of 10, 30, 50, 100, 150, 200 and 250 mTorr. The structural characteristics of the films were characterized using x-ray diffraction (XRD), X-ray reflectivity (XRR), Reflectivity High Energy Electron Diffraction (RHEED), and atomic force microscopy (AFM), and magnetic properties were measured using a commercial Physical Properties Measuring System (PPMS). New features presented here as compared to the recent previous studies^20^ include the analysis of the XRR data using the two-layer model which provided thickness and roughness of the intermediate and surface layers, detailed temperature and magnetic field dependence of the magnetization in zero-field-cooled and field-cooled modes, and analysis of the variation of the *T*_C_ with oxygen pressure in terms of oxygen vacancy density. Considering these factors, it is found that the 30 nm LSMO film grown at 150 mTorr with *T*_C_ ~340 K has the best overall characteristics such as the lowest roughness, and the best room temperature magnetic characteristics (highest saturation and remanent magnetization, and low coercivity). The observation of the negative remanent magnetization for zero field cooled samples and its dependence on the oxygen growth pressure is also reported. Details of the synthesis of these films along with their structural and magnetic characterization and discussion and analysis of the results are presented in the following pages.

## Growth and Structural Characterization of Films

### Unit-Cell-Controlled growth process

**Figure 1:**
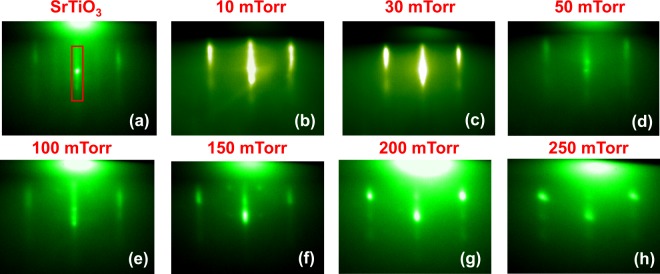
The RHEED diffraction pattern captured along the (001) direction after completion of the film growth before cooling down at various oxygen deposition pressures of 10, 30, 50, 100, 150, 200, and 250 mTorr, respectively.

**Figure 2:**
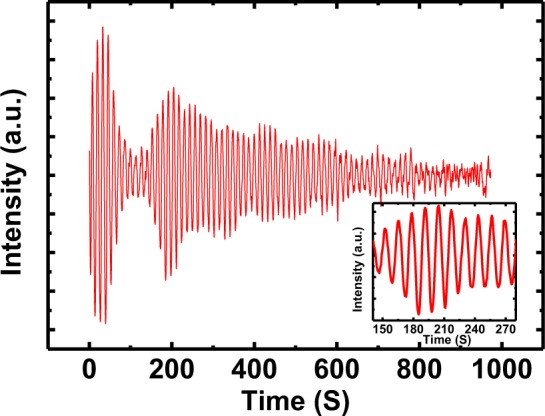
The RHEED oscillations of the LSMO film synthesized at oxygen deposition pressure of 10 mTorr.

The growth processes of all LSMO films on STO (SrTiO_3_) substrates were monitored *in-situ* with RHEED. Figure 1(a) shows the RHEED pattern taken along the (001) direction of a TiO_2_ terminated STO substrate prior to deposition at 750 °C in 100 mTorr oxygen pressure. We observed a very intense main specular spot compared to the two-sided spots which is clear indication of TiO_2_ terminated STO^24^. Similar types of features were also seen in STO substrates at other growth pressures (not presented here). Figure 1(b–h) presents the RHEED pattern taken along the (001) orientation collected right after the deposition of films on the substrates at different oxygen pressures of 10, 30, 50, 100, 150, 200, and 250 mTorr respectively. The presence of a sharp streaky line in the respective RHEED diffraction patterns on surface of LSMO films deposited at 10, 30, 50, 100, and 150 mTorr suggests two-dimensional (2D) growth with relatively smooth LSMO surfaces^25^. This streaky feature is changing to a streaky feature including spots with increasing oxygen pressure (for 200 and 250 mTorr), which reveals that the growth mode has changed from 2D to the combination of 2D and 3D growth. The intensity of RHEED pattern marked in red rectangular area (Fig. 1(a)) is observed during the growth of high-quality LSMO thin films. Clear RHEED intensity oscillations can be seen in Fig. 2, indicating 2D growth in layer-by-layer mode^24^. The oscillation of RHEED pattern for the deposition of a 80 unit-cells film grown in 10 mTorr is shown in Fig. 2 as representative after appropriate background correction. For this oxygen pressure, it takes 10 sec to deposit one layer of LSMO on the surface of the STO substrate; higher oxygen pressures increase deposition time. The 80 total peaks of the RHEED intensity pattern indicate the growth of 80 atomic layers of LSMO on the top of the STO substrate. The relatively smaller lattice mismatch observed between LSMO and STO helps this 2D growth maintain layer-by-layer mode. Damping in RHEED pattern is found during the growth of LSMO and can be accredited to the coexistence of layer-over-layer and step flow mechanisms of growth^26^. The amplitude goes back up as observed around 200 s, it is not common for the amplitude to rebound. The oscillation amplitude can increase likely due to an increase in step density^27,28^.

By lowering the oxygen growth pressure causes oxygen deficiency, creating more oxygen vacancies and/or defects in these films synthesized at 10, 30, 50 and 100 mTorr oxygen growth pressure. In the synthesis of the films deposited under higher oxygen partial pressures (> 200 mTorr), the energy of the laser ablated LSMO particles generally decreases after striking with the atoms of oxygen. Also, during the growth of the films under greater oxygen growth pressures, higher number of oxygen atoms are likely to be stuck on the surface of the substrate. When the laser ablated particles reach to the surface of the substrate, they must have to consume more energy to move the stuck atoms of oxygen to be deposited on the substrate. This will increase number of vacancies, and/defects contents of LSMO films grown above 200 mTorr. This suggests that there is an optimum growth pressure of oxygen for producing higher quality films.

### X-ray diffraction (XRD) studies

**Figure 3:**
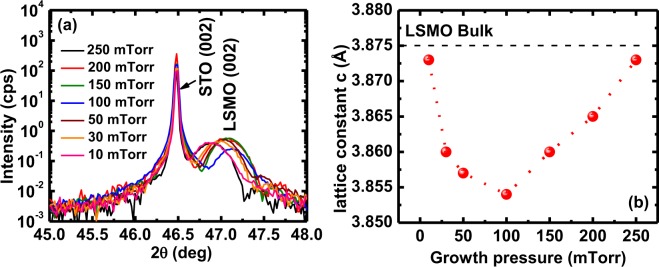
(**a**) The (002) XRD reflection, and (**b**) resulting calculated out-of-plane lattice constant {c(Å)} of the LSMO films grown under various oxygen deposition pressures of 10, 30, 50, 100, 150, 200, and 250 mTorr. The black dashed line (**b**) shows the bulk LSMO lattice parameter.

The phase purity, crystalline quality and other structural properties of the as-grown LSMO films deposited at several oxygen partial pressures were also investigated by X-ray diffraction technique (XRD). In large angle x-ray scans (20° to 80°, not shown here), all the thin films exhibit only diffraction peaks from the STO substrate and (00 *l*) pseudo-cubic reflections from LSMO, indicating that these films are highly oriented in nature. We did not notice any additional reflections that would be indicative of impurity or secondary phases confirming the phase purity of the thin films. Figure 3(a) depicts the *(002*) planes of all LSMO films grown on STO perovskite single crystalline substrates grown under various oxygen pressures (from 10 to 250 mTorr). It is clearly observed that (Fig. 3(a)), the XRD peaks move towards higher angle by decreasing of oxygen growth pressure down to 100 mTorr suggesting a decrease in lattice parameter, whereas from 50 mTorr the XRD peaks shift towards lower 2θ by lowering the oxygen growth pressure during the deposition process suggests an increase of out-of-plane lattice constants. The change in the out-of-plane lattice constant of all the films deposited at different oxygen growth pressures is plotted in Fig. 3(b). The increase of lattice parameter and decrease of the strain by lowering the oxygen growth pressure could be explained in terms of oxygen vacancies. Since oxygen ions are charged, their vacancy results in the neighboring cations to push apart due to Coulomb repulsion, which typically results in increasing the volume^29^. The other possible reason can be the decrease of stiffness in crystals with vacancies^30^. The lattice constants of LSMO films are smaller than the lattice parameter of bulk LSMO (~0.3876 nm) computed from XRD patterns^31^. This smaller value is expected for the out-of-plane direction, since the in-plane direction is increased to epitaxially match the substrate’s in-plane lattice parameter. The increase of lattice parameter by lowering the oxygen growth pressure indicates that the formation of oxygen vacancies in the material are responsible for changing the lattice parameter^32,33^. The increase of lattice parameter and decrease of the strain in the direction of higher growth pressures can be explained in terms of release of tensile strain due to increasing roughness. The roughness increases in the LSMO films deposited at higher growth pressure because of the combination of 2D and 3D growth as seen in RHEED patterns.

### X-ray reflectivity (XRR) studies

**Figure 4:**
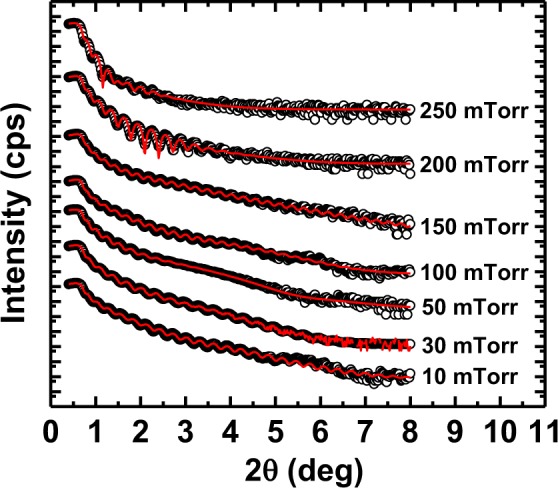
The experimental (open circles) and simulated (red curves) XRR data from LSMO films synthesized at various oxygen deposition pressures of ~10, 30, 50, 100, 150, 200, and 250 mTorr. The data and simulated curves for different samples are artificially displaced upwards by different amounts for reasons of clarity and identification.

To corroborate the thickness of LSMO thin films, small-angle XRR measurements were carried out. Modeling of the reflectivity data was performed using the GenX software to determine film thickness as well as the roughness of surface and interface. The experimental and simulated XRR curves for all the LSMO thin films deposited under different oxygen partial pressures are depicted in Fig. 4. As expected, interference fringes called Kiessig fringes were observed in the XRR spectra due to destructive and constructive interference between scattering from the surface of thin film and the film-substrate interface^34^. A two-layer model was found to fit well with the XRR data. The two-layer model has been used previously in LSMO and other films^34–37^. Based on the reflectivity analysis, we determined the thickness and the surface roughness of the individual layers and the results extracted from the fitting are presented in Table 1. We found that root mean square (RMS) roughness of the intermediate layer increases with increase of oxygen growth pressure except for 10 and 30 mTorr (see Table 1), whereas 10 and 150 mTorr films have small surface RMS roughness ~0.30 ± 0.01 and ~0.37 ± 0.01 nm (roughness of surface layer) respectively compared to other films. For this reason, we can see longer lived Kiessig fringes in 10 and 150 mTorr films, whereas less Kiessig fringes are observed in other films^34^. As the surface and interface roughen for the other thin films, the fringes broaden and vanish as the interference between scattering from the interface and surface loses coherence. Film thickness was calculated by considering the distance from the substrate/film interface where the scattering length density becomes half of the value of film^38^. The XRR results confirm that the films are uniform with smooth surfaces. The thickness of the films is 30 ± 3 nm, in agreement with the thickness value observed from RHEED data.

**Table 1:** The simulated XRR parameters of the LSMO films synthesized at various oxygen deposition pressures of ~10, 30, 50, 100, 150, 200, and 250 mTorr using the two-layer model (see Fig. 4).

Growth pressure (mTorr)	Thickness @ intermediate layer (nm)	Thickness @ surface layer (nm)	Film’s total thickness (nm)	Roughness @ intermediate layer (nm)	Roughness @ surface layer (nm)
250	26.47 ± 0.40	3.03 ± 0.16	29.5	1.82 ± 0.15	1.62 ± 0.25
200	24.53 ± 0.37	2.14 ± 0.42	26.67	1.61 ± 0.41	1.10 ± 0.08
150	30.32 ± 0.28	2.28 ± 0.20	32.59	0.88 ± 0.10	0.30 ± 0.01
100	30.28 ± 0.22	1.57 ± 0.14	31.85	0.52 ± 0.16	0.39 ± 0.02
50	26.35 ± 0.81	2.44 ± 0.05	28.79	0.30 ± 0.11	0.42 ± 0.02
30	24.30 ± 0.55	1.91 ± 0.21	26.21	0.74 ± 0.29	0.50 ± 0.22
10	25.69 ± 0.30	2.88 ± 0.26	28.57	0.60 ± 0.24	0.37 ± 0.01

## Magnetic Properties of LSMO Thin Films

**Figure 5:**
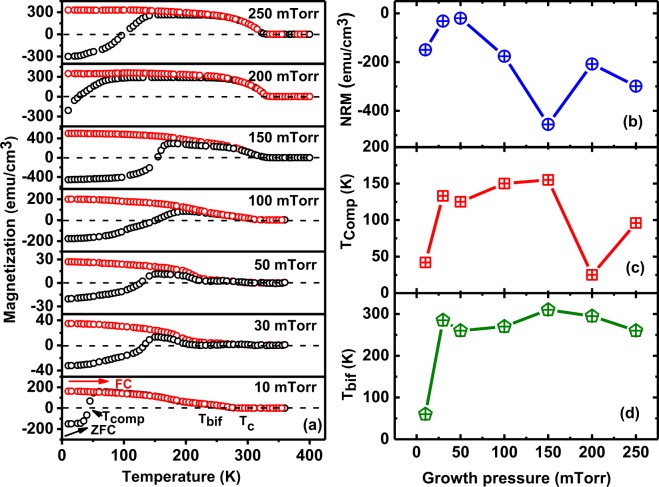
(**a**) *M* versus *T* (both ZFC and FC) with an static applied *H* of 50 Oe, (**b**) negative remanent magnetization (NRM) at 5 K, (**c**) compensation temperature (*T*_comp_), and (**d**) bifurcation temperature (*T*_bif_) of the LSMO thin grown under various oxygen deposition pressures of 10, 30, 50, 100, 150, 200, and 250 mTorr.

**Figure 6:**
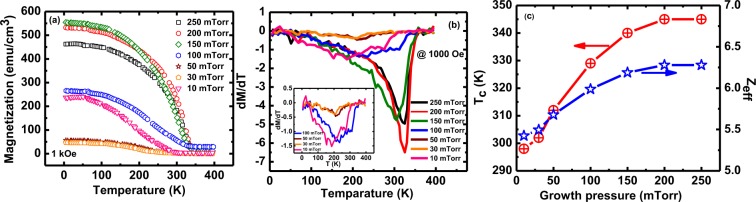
(**a**) The magnetization versus temperature (FC), (**b**) the computed d*M*/d*T*, (**c**) Curie temperature (*T*_C_) (left panel) and *Z*_eff_ (right panel) of the LSMO films synthesized at various deposition pressures of ~10, 30, 50, 100, 150, 200, and 250 mTorr under static applied *H* of 1000 Oe.

**Figure 7:**
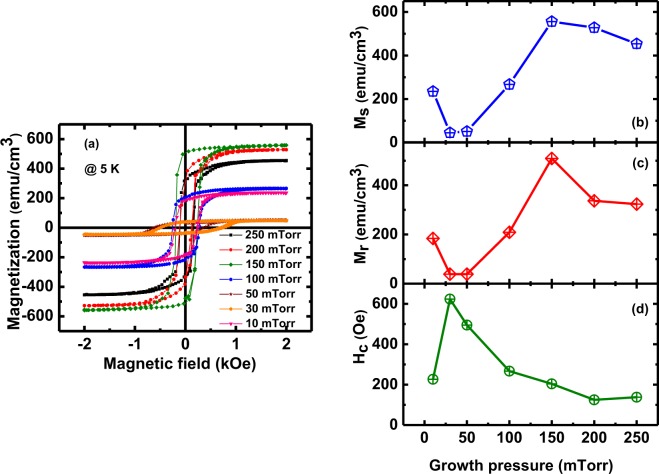
(**a**) The magnetic field dependence of magnetization (*M*-*H* loops); Variation of (**b**) *M*_s_, (**c**) *M*_r_, and (**d**) *H*_c_ of the LSMO films with deposition pressures of 10, 30, 50, 100, 150, 200, and 250 mTorr.

**Figure 8:**
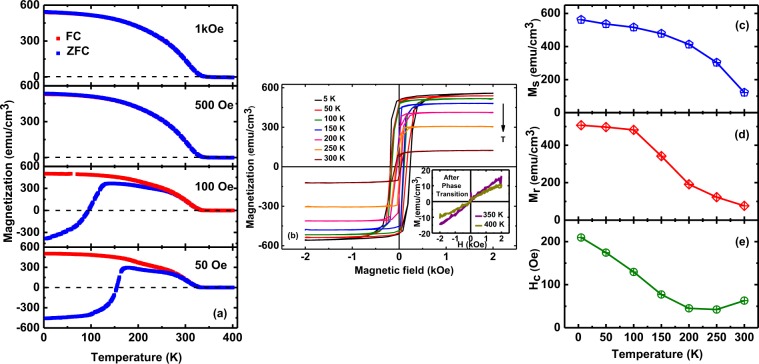
Plots of (**a**) *M* versus *T* (**b**) *M* versus *H* (**c**) *M*_s_ versus *T* (**d**) *M*_r_ versus *T*, and (**e**) *H*_c_ versus *T* for the LSMO thin films grown at an oxygen deposition pressure of 150 mTorr.

We carefully examined the magnetic behaviour of all LSMO films synthesized at different oxygen partial pressure by performing the magnetic field and temperature dependence of dc-magnetization measurements (Figs. 5–8). All measurements of the magnetization (*M*) reported here were performed in the in-plane mode and the *M* values of all films are suitably corrected as STO substrates exhibit diamagnetic behavior which contribute noticeable *M* at higher fields. Magnetization measurements as a function of temperature of all the films were performed in zero-field-cooled (ZFC) and field-cooled (FC) mode by applying static magnetic fields of 50 and 1000 Oe in a wide range of temperature (5 to 400 K). The details of measurement protocol is mentioned in our previous paper^39^. Fig. 5(a) depicts the ZFC and FC curves measured under *H* = 50 Oe. Magnetization decreases with the increase of temperature for the FC mode, as is typical in ferromagnets. In the ZFC curve, we observed *M* < 0 at low temperature measured in the low field of 50 Oe. By increasing temperature, *M* switches sign. Therefore, we observed negative remanent magnetization (NRM)^35^ for *T* < 150 K for all LSMO thin films synthesized at different oxygen partial pressures. Magnetization increases with an increase of temperature and then decreases above bifurcation temperature *(T*_bif_). The NRM effect appears in a material when the direction of *M* is reverse to the direction of the applied *H* to that material^35^. NRM has been already observed and reported by our group in LSMO thin films grown in 100 mTorr^35,39^. Lee *et al*. have also observed NRM in LSMO films via X-ray magnetic circular dichroism (XMCD) technique^34^. NRM in LSMO/PZT heterostructures has been studied by Sandra *et al*.^40^. This NRM has been observed only when the measuring field is less the coercive field (*H*_c_) since for applied *H* > *H*_c_ only positive values of *M* are observed in ZFC. This observed NRM is attributed to the coexistence of magnetically inhomogeneous regions consisting of spin clusters of a lower oxidation state and the ferromagnetic order in LSMO thin films^35,39,41–43^. The growth pressure dependence of NRM is plotted in Fig. 5(b), which shows that 150 mTorr sample has the highest NRM among all samples. The temperature at which magnetization vanishes and changes sign (from negative to positive in the ZFC curve and from positive to negative in the FC curve) is known as the compensation temperature *(T*_comp_) shown in Fig. 5(c)^39^. *T*_comp_ increases monotonically with an increase of growth pressure up to 150 mTorr and then decreases. We have also plotted the *T*_bif_ in Fig. 5(d) as a function of growth pressure; here *T*_bif_ is the temperature at which the FC curve bifurcates from the ZFC curve^44^.

Figure 6(a) shows the FC curves measured in applied *H* = 1 kOe; it is evident that all the thin films synthesized at different growth pressures exhibit ferromagnetic behavior (as also confirmed in the next figure) since as in ferromagnets, *M* gradually decreases with increase of temperature up to the Curie temperature (300–345 K here) (Fig. 6(a)). To investigate the nature (sharpness) of the magnetic phase transition, the temperature dependence of computed *dM/dT* (the temperature derivative of the magnetization curves from the plots of Fig. 6(a), are depicted in Fig. 6(b)^18^. The *dM/dT* curves reveal the broad feature of the transition for the films deposited at lower oxygen growth pressure (10, 30, 50 and 100 mTorr) and with an increase of oxygen deposition pressure, the transitions become sharper. These results suggest that by lowering the oxygen growth pressure will increase the defects as oxygen vacancies in the thin films, and thus encourages the oxygen nonuniformity and thus affects the nature of the phase transition^18^. The ferromagnetic-paramagnetic transition temperature *(T*_*C*_) plotted with respect to their respective growth pressure in the left panel of Fig. 6(c) shows that *T*_*C*_ decreases with decrease of oxygen growth pressure. Magnetic behavior of the manganites are generally depend upon the double-exchange interaction among d-orbitals magnetic ions (Mn^3+^ and Mn^4+^) by transferring the charge from magnetic Mn^3+^ and Mn^4+^ ions via oxygen atoms^32^. Oxygen vacancies increases when the films are deposited at lower oxygen growth pressure, which break the exchange mechanism and lead to higher spin disorderness hence decreases *T*_C_. The highest *T*_*C*_ = 345 K observed for the films synthesized at 200 mTorr and 250 mTorr oxygen pressures is somewhat less than *T*_*C*_ = 369 K observed for bulk LSMO samples; this is likely due to thickness dependence of *T*_*C*_ since the films grown in this work have a thickness of about 30 nm.

Magnetic hysteresis loop measurements are the response of *H* dependence of *M* which depends on composition, size, structure, imperfections of a material and direction of *H* with respect to the easy axis. As discussed in our earlier paper on the LSMO film of thickness ~7.6 nm^35^, the quantities of interest here are the remanent magnetization (*M*_*r*_) for *H* = 0, saturation magnetization (*M*_*s*_) and the coercive field (*H*_*c*_). To remove *M*_*r*_, it is necessary to apply *H* in the reverse direction to demagnetize the material known as the coercive field. Hysteresis loop are due to the presence of domains, their pinning by defects, and changes in the domain size as *H* is increased. At saturation, the material has a single domain with magnetization parallel to *H*^35^. Figure 7(a) shows the hysteresetic behavior of LSMO films synthesized at various oxygen growth pressure recorded at 5 K up to ± 2 kOe. All films exhibit well saturated hysteretic behavior above 1000 Oe, which is the typical signature of ferromagnetic materials, irrespective of deposition pressure. The shapes of the curves are nearly rectangular because the magnetization lies in the plane of the films and *H* is applied parallel to the film planes. The initial magnetization rapidly grows in the low-field regime (<1000 Oe); however, at high fields, the magnetization growth becomes weaker and tends to saturate. The *M*_s_, *M*_r_, and *H*_c_ plotted against the growth pressure are shown in Fig. 7(b–d) respectively. Ignoring the data for the 10 mTorr sample for the time being, it can be seen that *M*_s_ and *M*_r_ effectively increase and *H*_*c*_ decrease monotonically with the increase of oxygen growth pressure (Fig. 7(b–d)) with only minor changes occurring above 150 mTorr. These observations can be explained in terms of the reduction in the oxygen vacancies with increase in growth pressure. At optimal conditions, there is a mix of Mn^3+^ and Mn^4+^ cations, and this mixture is important for the double exchange mechanisms. We have observed that as more oxygen vacancies are introduced, the number of Mn^4+^ cations decrease which initially lowers the magnetization. However, we have to able to increase the oxygen vacancies further while maintaining excellent crystal structure and eventually the magnetization improves. For 10 mTorr sample, the ratio of Mn^2+^/Mn^3+^ increases with the increase of oxygen vacancies density which results in the improvement of the double exchange interaction^32^. Hence, we observed the increase of *M*_s_ at 10 mTorr. The *H*_*c*_ of thin films grown at 30 mTorr is found to be higher compared to the thin films grown at other investigated pressures (Fig. 7(d)). The *H*_*c*_ systematically decreases with the increase of the growth pressure except for 10 mTorr. With the increase of oxygen growth pressure, point defects and structural disorder decreases, which decreases the *H*_*c*_^45,46^.

We further analyzed the magnetic behavior in detail of LSMO films grown at 150 mTorr as these films exhibit the highest magnetization (Fig. 8). NRM was observed for the ZFC case when the measuring field *H* = 50 and 100 Oe which is less the coercive field *(H*_c_); for higher *H* = 500 and 1 kOe *(H* > *H*_c_*)*, only positive values of *M* are observed in ZFC curves. We observed very small bifurcation in the FC-ZFC curve from *M* vs. *T* data measured at 500 and 1 kOe. The occurrence of this small bifurcation behavior at high applied field might be due to the application of external magnetic fields larger than the coercive field *(H*_c_*)*^47^. For *H* = 500 Oe and 1 kOe, magnetization slowly decreases with increasing of temperature till it disappears above ~340 K (Fig. 8(a)). The estimated ferromagnetic-paramagnetic *T*_C_ is found to be 345 (+/−5) K. Figure 8(b) shows the *M-H* loops of the film grown at 150 mTorr at different temperature. The magnetization disappears above the phase transition (inset of Fig. 8(b)). The *M-H* loops recorded above *T*_*C*_ clearly show paramagnetic behavior which corroborate the observation of magnetic phase transition (inset of Fig. 8(b)). The *M*_s_ and *M*_r_ of these films at 5 K is observed to be ~562 and 507 (+/−5) emu/cm^3^ respectively and gradually decreases with increase of temperature whereas the *M*_s_ and *M*_r_ are ~122 and 76 (+/−5) emu/cm^3^ at room temperature (Fig. 8(c,d)). The observed gradual decreases in *H*_c_ with increase of temperature is consistent with observations in other ferromagnetic thin films (Fig. 8(e))^48^.

The data presented here can be interpreted by realizing that the magnetic characteristics of LSMO films are strongly influenced by the number of Mn nearest neighbors (Z). For ideal bulk LSMO (thick films), the value of *Z* for Mn is 6 as each Mn is surrounded by an oxygen octahedral containing 6 oxygen ions^39^. To further explore the effect of oxygen growth pressure on the effective nearest neighbors *(Z*_eff_*)*, we have calculated *Z*_eff_ from the molecular-field based equation:

3 $${Z}_{{\rm{eff}}}=3{{\rm{T}}}_{{\rm{c}}}/2({\rm{J}}/{{\rm{k}}}_{{\rm{B}}})\,{\rm{S}}({\rm{S}}+1)$$

here *T*_C_ is the magnetic transition temperature, *J/k*_B_ is the exchange constant and *S* is the effective spin^39^. Assuming that *J/k*_*B*_ and *S* do not change with oxygen growth pressure, the observed change in *T*_*C*_ with oxygen growth pressure can be directly related to changes in *Z*_eff_ since an oxygen vacancy will disrupt the exchange coupling between the nearest neighbors. Consequently, in the right panel of Fig. 6(c) we have also plotted *Z*_eff_ versus oxygen growth pressure. With decreasing oxygen growth pressure oxygen vacancies increase, hence the effective number of nearest neighbors with which the spins are coupled decreases which results in the decrease of *Z*_eff_ and hence the magnetization and transition temperature are lowered, as discussed in the above sections.

Here, we noticed that the defects/oxygen vacancies concentration increases both by increasing and reducing in oxygen partial pressure during growth. The increase or decrease of oxygen vacancies and defects strongly affect the MnO_6_ octahedron structures those determine the physical functionalities of the LSMO films^22+^. For films with few oxygen vacancies, the optimal magnetization occurs at 150 mTorr at low temperature. At pressures above our optimal value, the surface roughness increases drastically. Removal of the oxygen linkers (which occurs at lower oxygen pressures) will have a significant effect on the magnetization.

## Conclusions

Highly epitaxial and high quality LSMO films were synthesized on STO single crystalline substrates at different oxygen partial pressure by PLD. By monitoring the *in-situ* growth with RHEED, we find that LSMO can grow epitaxially on perovskites STO substrate for all growth pressures. All the thin films synthesized at different oxygen growth pressures are found to be very smooth with low roughness; the LSMO films grown at 150 mTorr exhibits lowest roughness among all thin films. The magnetic properties of all LSMO films synthesized at different oxygen deposition pressures are correlated with the oxygen defects/vacancies. The thin films synthesized at lower oxygen growth pressure exhibit lower magnetic *T*_*C*_ and a diffuse type of phase transition. We observed the systematic variation of the magnetic properties with change in the oxygen growth pressure. Thin films grown at 150 mTorr exhibit the lowest roughness, highest saturation and remanent magnetization with *T*_C_ ~340 K. The existence and growth pressure dependence of NRM of the above-mentioned thin films when cooled in zero-magnetic field is another important result. All these LSMO thin films exhibit large *M*_*s*_, well defined ferromagnetic hysteretic characteristics above RT, and might be potentially utilized in future nanoscale nonvolatile memory, spintronics, and many multifunctional devices.

## Methods

**Figure 9:**
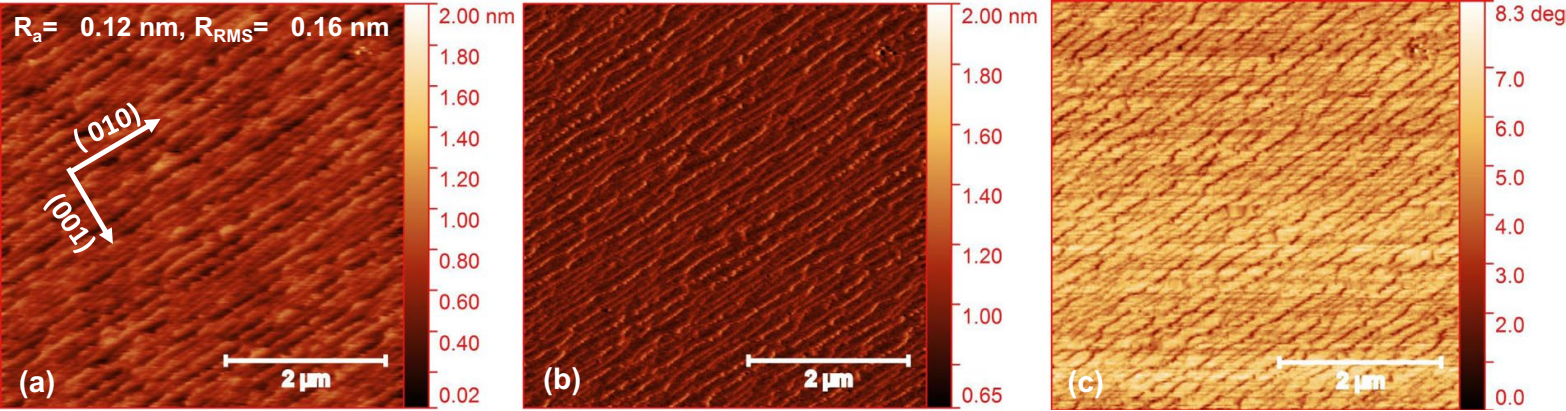
The atomic force micrographs of (**a**) height, (**b**) amplitude, and (**c**) phase of treated STO substrate (discussion is in method section).

We have chosen STO (001) substrates for the synthesis of highly epitaxial LSMO thin films with minimal strain as the lattice parameter of STO (a = 3.905 Å) matches well with the lattice parameter of LSMO (a = 3.876 Å). The miscut between the substrate surface and crystal plane is less than a half degree i.e. (001) +/−0.5 deg. One of the most important requirements for the growth of epitaxial film on atomically smooth surface of single-termination (TiO_2_ terminated) STO substrate having a step height of a unit cell^15^. To get atomically smooth step-and-terrace configurations having single c termination, the substrates were first ultrasonically cleaned in acetone, isopropanol, and deionized water for 5 mins then thermally treated at 1100 °C × 2 hours. This is the typical substrate treatment process^49^. After cooling down to room temperature, substrates were again ultrasonically cleaned into deionized water for 5 mins and again annealed at 1100 °C × 2 hours. STO (001) substrates of singly B-site TiO_2_ terminated were prepared after this process. In some cases, mixed termination was seen, perhaps due to reduced quality of STO substrates. These behaviors were seen from the phase contrast of the AFM images. These substrates were treated with deionized water again, followed by the annealing process, after which no significant SrO termination has been detected. The Atomic Force Micrographs of treated STO substrate on a scan size of 5 × 5 μm^2^ shows an atomically smooth surface with clear steps as shown in Fig. 9.

Thin films of LSMO were fabricated on atomically flat and chemical terminated (TiO_2_ terminated) SrTiO_3_ (001) substrates using PLD (commercial Neocera PLD system) with an excimer laser (KrF, λ = 248 nm). To explore the effects of oxygen deposition pressure dependence of physical properties of LSMO, the oxygen growth pressure was systematically changed from 10 to 250 mTorr keeping the substrate temperature fixed ~750 °C which was found to be the optimized growth temperature during all depositions. The laser energy density was maintained ~3 J/cm^2^ with a frequency of 5 Hz for all thin films. The distance between the substrate and target was kept fixed ~10 cm. Immediately after the deposition, oxygen ambient pressure was changed to 450 mTorr inside the PLD chamber to prevent oxygen loss while cooling and improve the oxidation quality. *In-situ* Reflection High Energy Electron Diffraction (RHEED) had been utilized to inspect the entire growth of the films. The RHEED images were taken using a CCD camera at the end of the growth. Structural properties of all thin films such as roughness, thickness, and density were computed from X-ray Reflectivity (XRR) measurements. XRR measurements of all these above-mentioned films were performed using a Bruker D8 Discovery X-ray Diffractometer (XRD). To check the orientation and phase purity of all films, X-ray diffraction (XRD) measurements were performed by CuKα radiation (λ = 1.5405 Å). Since the magnetic behavior of the LSMO films also depend on their thickness, the thickness of all the films was kept at about the same (~30 nm) in order to focus only on changes with change in oxygen pressure. *T* dependence of *M* for all the films was measured in a wide temperature range (5–400 K) by a Physical Property Measurement System (PPMS) by Quantum Design. The magnet coil was demagnetized before each measurement so that the residual magnetic field is reduced to zero. Samples were also demagnetized by heating up the sample above their magnetic ordering temperature.
